# Nitric Oxide Sustains IL-1β Expression in Human Dendritic Cells Enhancing Their Capacity to Induce IL-17–Producing T-Cells

**DOI:** 10.1371/journal.pone.0120134

**Published:** 2015-04-08

**Authors:** Carolina Obregon, Lukas Graf, Kian Fan Chung, Valerie Cesson, Laurent P. Nicod

**Affiliations:** 1 Pneumology Service, Centre Hospitalier Universitaire Vaudois and University of Lausanne, Lausanne, Switzerland; 2 Clinic and Polyclinic of Pneumology, University Hospital of Bern, Bern, Switzerland; 3 Airways Disease Section, National Heart & Lung Institute, Imperial College, London, United Kingdom; University Medical Center Freiburg, GERMANY

## Abstract

The role played by lung dendritic cells (DCs) which are influenced by external antigens and by their redox state in controlling inflammation is unclear. We studied the role played by nitric oxide (NO) in DC maturation and function. Human DCs were stimulated with a long-acting NO donor, DPTA NONOate, prior to exposure to lipopolysaccharide (LPS). Dose-and time-dependent experiments were performed with DCs with the aim of measuring the release and gene expression of inflammatory cytokines capable of modifying T-cell differentiation, towardsTh1, Th2 and Th17 cells. NO changed the pattern of cytokine release by LPS-matured DCs, dependent on the concentration of NO, as well as on the timing of its addition to the cells during maturation. Addition of NO before LPS-induced maturation strongly inhibited the release of IL-12, while increasing the expression and release of IL-23, IL-1β and IL-6, which are all involved in Th17 polarization. Indeed, DCs treated with NO efficiently induced the release of IL-17 by T-cells through IL-1β. Our work highlights the important role that NO may play in sustaining inflammation during an infection through the preferential differentiation of the Th17 lineage.

## Introduction

DCs recognize foreign antigens through various pathogen-associated molecular patterns (PAMPs), as well as damage-associated molecular patterns (DAMPs), which are associated with tissue and cell damage during infection. Depending on the antigen, DCs are able to modulate immunity or tolerance [1, 2]. However, DCs are not alone in undertaking this task as their function depends on their localization and is highly modulated by factors produced by stromal cells and epithelial cells (ECs). In the lung, ECs are important cells situated at the interface with the external environment and, during an infection, they form an early critical component of the innate immune defence mechanism in the airways through their ability to produce high levels of nitric oxide (NO) [3]. The production of NO^.^ is catalysed by nitric oxide synthases from L-Arginine. The inducible isoform, inducible nitric oxide synthase (iNOS), is constitutively present in the airway epithelium, and is markedly upregulated in inflammatory conditions such as asthma [3, 4] and chronic obstructive pulmonary disease (COPD) [5, 6]. However, its role in inflammation has raised interesting controversies. For example, during DC maturation both an increased and a reduced release of IL-12 by NO^.^ has been reported [7–9]. In mouse model, NO^.^ has also been shown to be a co-factor that can regulate differentiation signals on T-helper cells. Depending on the prevailing cytokine environment such as TGF-β and IL-6, NO^.^ is able to antagonize Th17 differentiation [10, 11].

IL-17 is a pleiotropic cytokine that can stimulate host defences during bacterial and fungal infections[12] as well as increasing inflammation, which results in tissue damage and autoimmune responses. There is good evidence that IL-17A expression is increased in asthma and that IL-17A may play a role in steroid-resistant asthma [13]. IL-17 produced from γδT cells have been shown to mediate the resolution of allergic airway inflammation and airway hyperreactivity in a murine model of allergic inflammation [14]. In COPD, IL-17 is found upregulated in the bronchial submucosa with the presence of TH17 cells and CD8 IL-17 secreting cytotoxic T cells [15].

In chronic lung diseases such as COPD and more so in asthma, NO^.^ has been associated with disease progression [16]. However, no clear correlations have been established between inflammatory events and lung disease status or severity; additionally, a better understanding of the factors that involve high susceptibility and severity to infections in these patients is needed [17, 18]. Because IL- 17 is a key cytokine in asthma and COPD as described above, we therefore asked to what extent was nitric oxide able to modulate IL-17 production by T cells. In order to unravel the role of NO^.^ during inflammation, human DC were pre- treated with NO^.^ prior to addition of lipopolysaccharide (LPS), and the innate and adaptive responses were analysed.

In this paper, we showed that NO^.^ can change the pattern of cytokine release by LPS-matured DCs, which was dependent on the concentration of NO^.^ used, as well as on the timing of the addition of NO^.^ to the DCs during their maturation process. The major outcome of our study is the novel demonstration that NO^.^ sustains the expression and release of IL-1β in matured DCs, thus enhancing their capacity to induce IL-17 production by T-cells.

## Material and Methods

### Dendritic cell preparation and stimulation

PBMCs were isolated from buffy coats of healthy donors. In accordance with the Cantonal Ethics Committee of the Canton of Vaud (Vaud-Switzerland), written consent from the donors was obtained by the Lausanne blood transfusion center, the donors agreed that after absolute anonymity that certain components of their blood be used for medical research purposes. PBMCs were isolated by Ficoll Paque density gradient centrifugation. Monocytes (Mo) were prepared as described [19, 20] and were characterized by high expression of CD14 (more than 88%). Differentiation of DCs from monocytes was performed as originally described by Sallusto and Lanzavecchia, by culturing cells in the presence of granulocyte-macrophage colony-stimulating factor (10 ng/ml) and interleukin-4 (10 ng/ml) for 5 days [21]. At day 5, DCs were stimulated with lipopolysaccharide (LPS, DIFCO E. coli 055:B5) and nitric oxide (NO) donor, DPTA NONOate. Two different experimental protocols were performed to determine the effect of dose and time of stimulation using NO^.^ during LPS-induced maturation of DCs. For concentration-dependent experiments, cells were stimulated with different concentrations of DPTA NONOate added 10 minutes before LPS stimulation (100ng/ml). For time-dependent experiments, DPTA NONOate (0.6mM) was added 10 min, 1 or 5 hours before LPS maturation. To demonstrate the specificity of NO, cells were cultured with Carboxy-PTIO (5μM) 30 min before DPTA NONOate (0.6mM). For some experiments DPTA NONOate was resuspended in electrolyzed water (EW) and compared to the usual solution of DPTA NONOate resuspended in RPMI. After 1 h of incubation with DPTA NONOate cells were stimulated with LPS (100ng/ml). Electrolyzed water was obtained by electrolysis of deionized water (Milli-Q plus 185 model from Millipore, Zug, Switzerland) with 0.1g/L NaCl, with 1.5V for 5 min as previously described [22]. Release of NO was detected by measuring the fluorescence emission intensity of a specific NO indicator, DAF-FM diacetate (5μM) (Molecular Probes, USA). Supernatants were collected and stored at -80°C. Cells were immediately processed to measure the apoptosis rate and phenotypic changes by flow cytometry.

### Cytokine measurements

IL-17, IL-6, IL-1β IL-12p70, 12p40, IL-10, TNF-α and IL-13 cytokines were measured in the Luminex Bio-Plex 200 System (Bio-Rad, USA), using Bio-Rad kits according to the manufacturer’s instructions. IL-23 was measured with a commercial ELISA kit (Biotest, France).

### Flow cytometry

DC differentiation was determined by flow cytometry analysis using fluorescent-labelled monoclonal antibodies to CD86 (IgG1), CD80 (IgG1), and CD83 (IgG1), all FITC labelled (R&D), and isotype controls for IgG1-FITC. Delta MFI was defined as the MFI ratio of specific markers and isotype controls. Apoptosis and necrosis control was performed using Annexin-V-FITC and Propidium Iodide Staining (PI), respectively, according to the manufacturer's instructions.

### Mixed Lymphocyte Reaction (MLR)

DCs were stimulated at specific time-points with DPTA NONOate (0.6mM) and LPS (100ng/ml). T-cells used for allogenic MLR were isolated using Pan T-cell Isolation Kit II (Miltenyi Biotec, Germany). Isolated T-cells were added to washed DCs at DC:T-cell ratios of 1:10, 1:50 and 1:100. Cells were incubated for 6 days. For proliferation analysis, cells were pulsed for the last 18 h with 0.5 μCi of (^3^H)thymidine. Otherwise, supernatants were collected and stored at -80°C until required for cytokine measurement. Neutralizing experiments, were performed using the following neutralizing antibodies: IL-1β (CRM56, Ebioscience) used at 10μg/ml, IL-6R (Tocilizumab-Roche) (humanized IgG1) used at 10μg/ml and IL-23 (B-Z23, Diaclone) used at 10μg/ml.

### Real-time quantitative RT-PCR

Total RNA was extracted with the RNeasy mini kit (Qiagen Inc.) according to the manufacturer's instructions. Reverse transcription and PCR were performed using high capacity RNA-to-cDNA kit (Applied Biosystems). Transcripts were quantified by real-time quantitative PCR on an ABI PRISM 7900HT (Applied Biosystems) with Applied Biosystems predesigned TaqMan Gene Expression Assays and reagents, according to the manufacturer's instructions. The following probes were used (identified by Applied Biosystems assay identification number): TaqMan assay β-actin: 4326315E; IL1β, Hs01555413_m1; IL12A, Hs01073447_m1; IL12B, Hs01011518_m1; IL23A, Hs00372324_m1; IL6, Hs00985639_m1; CASP1, Hs00354836_m1. For each sample, mRNA abundance was normalized to the endogenous control β-actin using ΔΔCT values, and is expressed relative to control conditions.

### Statistical analysis

Results are expressed as means ± SEM. Comparisons between two groups were assessed by the Student's t test. The statistical significance was determined using the Holm-Sidak method. This analysis concerns the comparison the group of Caspase-1 inhibitor II with the respective group treatment andthecomparison the group of cells treated with LPS alone, with the group of cells treated with NO 30 min before LPS. Multiple group comparison was determined by one way analysis of variance followed by Bonferroni multiple comparisons test and was applied to the rest of data set analysis. p-values less than 0.05 were considered statistically significant. All statistical analyses were done with GraphPad Prism software (GraphPad Software, Inc., La Jolla, CA, USA).

## Results

### DPTA NONOate modulates cytokine release in a dose-dependent manner

**Fig 1:**
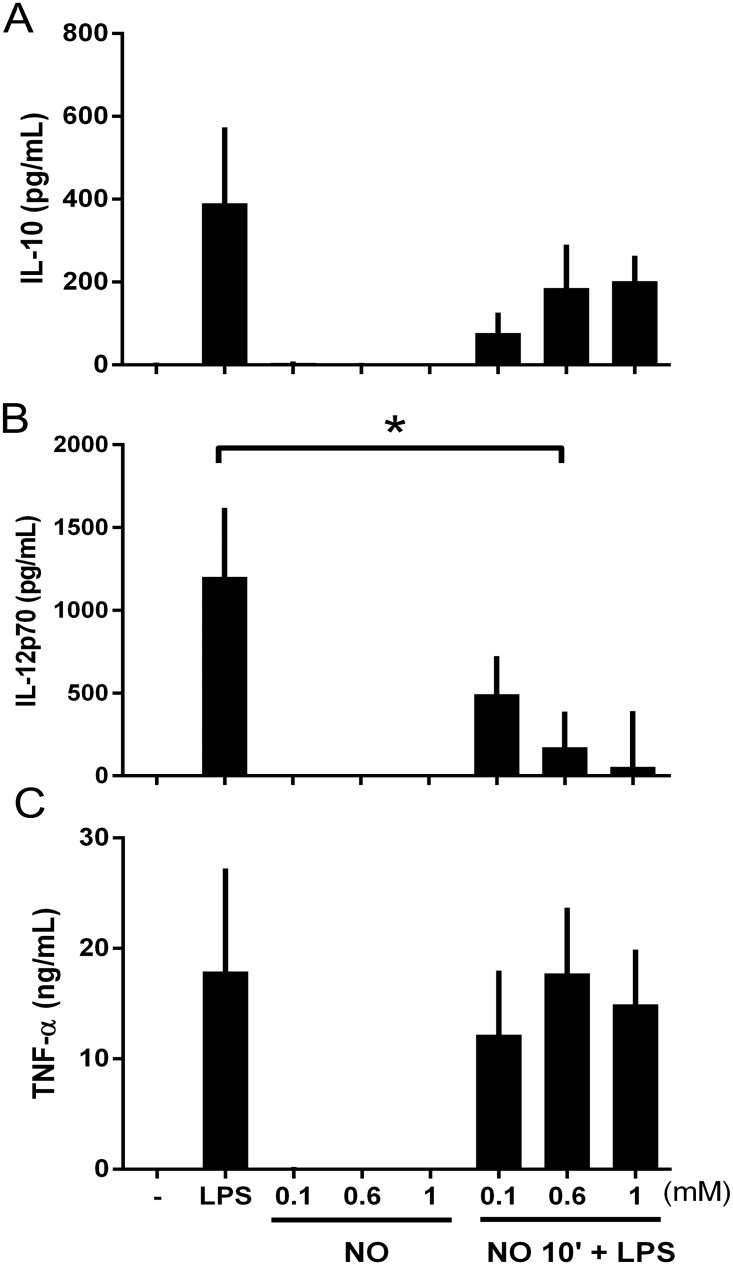
Cytokine release is dependent on NO^.^ concentration. DCs were stimulated for 23 h with different concentrations of DPTA NONOate 10 minutes before maturation with LPS (100ng/ml). Cytokines were measured using Luminex system as described in Material and Methods: (A) IL-10, (B) IL-12p70 and (C) TNF-α. Data are expressed as means ±SEM of 6 independent experiments. *P < 0.05.

To determine whether NO^.^ is able to modulate the release of cytokines from matured DCs, and therefore influence initiation and development of inflammation towards a pro-inflammatory or anti-inflammatory state, we used the NO donor, DPTA NONOate, which is able to release NO^.^ with a half-life of 5h at neutral pH. For these experiments, DPTA NONOate was added at different concentrations to immature DC cultures 10 min before the induction of maturation with LPS. Concerning the concentration of LPS, 100 ng has shown to be sufficient to stimulate DCs, inducing low cell death (apoptosis/necrosis) (S1 Fig). As already shown, LPS-matured DCs released a mixed pattern of Th1 and Th2 cytokines with high amounts of IL-10, IL-12 and TNF-α. Under our co-stimulatory conditions of DPTA NONOate and LPS, when a high concentration of DPTA NONOate was added before DC maturation, the release of TNF-α, as well as IL-10, was non-significantly modulated (Fig. 1A and C). On the other hand, the release of IL-12 was inhibited with DPTA NONOate at 0.6mM (Fig. 1B). These results show that NO^.^ act predominantly on IL-12 release and may modulate an inflammatory or anti-inflammatory process depending on the concentration used.

### DPTA NONOate abrogates IL-12 release at early time-points

**Fig 2:**
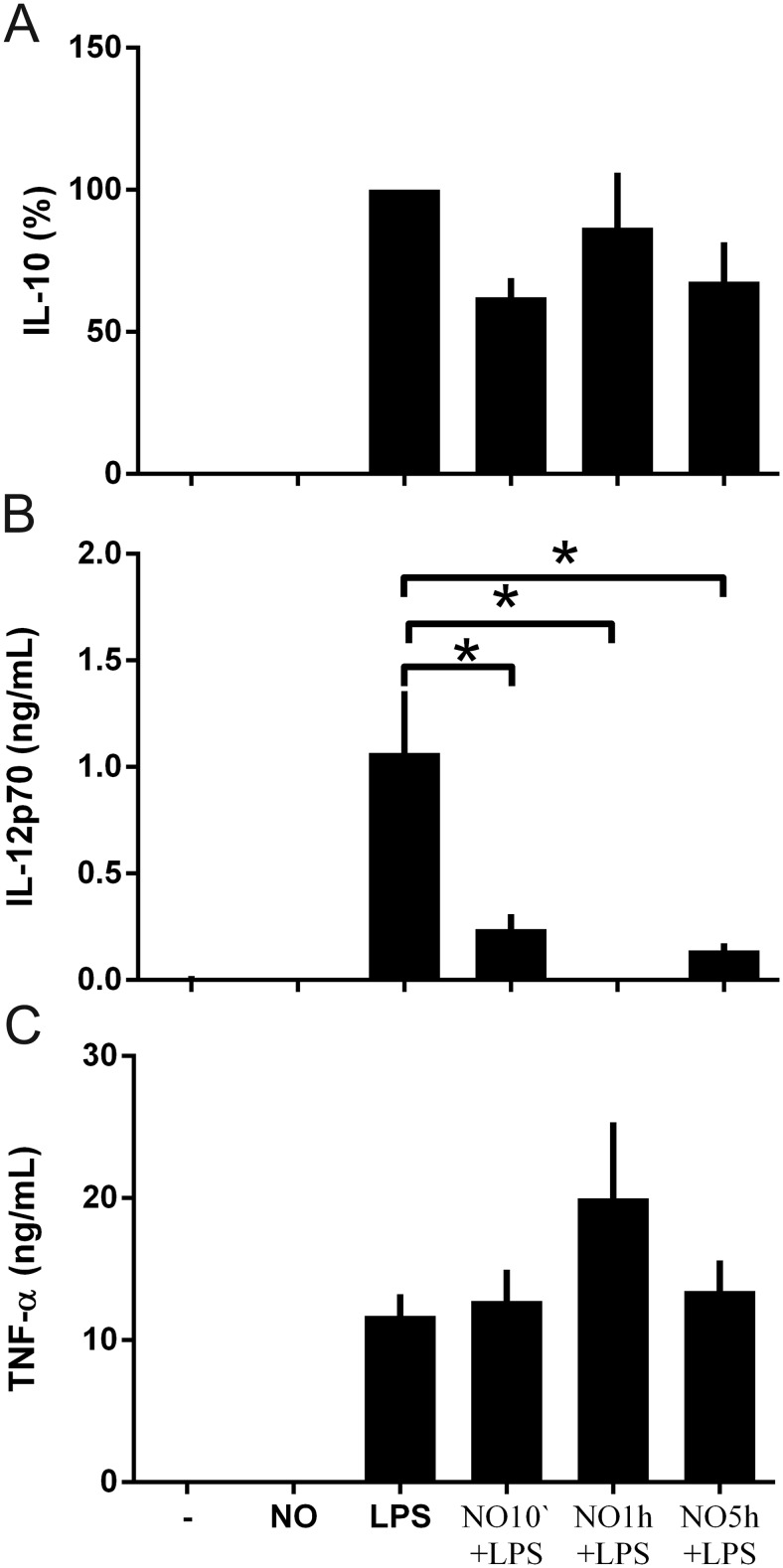
NO^.^ inhibits the release of IL-12 at early time-points. DCs were stimulated with DPTA NONOATE (0.6mM) 10 min, 1h and 5h before maturation with LPS (100ng/ml). The secretion of cytokines was analyzed using the Luminex system: (A) IL-10 (Due to a high variability of the IL-10 production the values were normalized to the levels of LPS %), (B) IL-12p70 and (C) TNFα. Data are expressed as means ±SEM of 11 independent experiments. *P < 0.05.

We next determined whether the duration of exposure to NO^.^ before DC maturation can modulate the pattern of cytokine release by DCs. A concentration of 0.6mM was used for further experiments, since the toxicity of DPTA NONOate is low, with the ability to prevent apoptosis and spontaneous necrosis (S1 Fig.) and DPTA NONOate was also able to induce significant changes in cytokine release. DCs were stimulated with DPTA NONOate for 10 min, 1 or 5 hours before maturation with LPS to determine if an extended exposure to NO^.^ was able to change the profile of cytokine release. When DCs were exposed to DPTA NONOate, the release of IL-12p70 and IL12-p40 was significantly reduced after only 10min incubation, but the inhibition was observed over the 5h period of incubation, compared to LPS-matured DCs (Fig. 2B and S2 Fig). DPTA NONOate did not modulate the release of IL-10 and TNF-α at any time-point (Fig. 2A and C). Thus, a short time pre-exposure to NO donor of 10 min before maturation with LPS was sufficient to convert DCs into an inhibitory role, modulating the inflammation induced by LPS, and decreasing the levels of IL-12. However, the 1- and 5-hour time periods were used for further experiments, since we considered it important that DCs should be exposed to NO^.^ over long periods of time and because the strongest cytokine modulation was observed at these time-points.

### DPTA NONOate-treated DCs increase the release of the pro-Th1 cytokine INF-γ and pro-Th17 cytokine IL-17

Based on the fact that NO^.^ inhibits the release of some pro-inflammatory cytokines such as IL-12, we asked whether NO^.^ could modulate the expression of co-stimulatory molecules, and hence the alloantigen-presenting capacity, or induce a specific T-helper polarization. The expression of CD86, CD80 and CD83 on DCs, co-stimulated with DPTA NONOate and LPS, was analyzed by flow cytometry (S3 Fig.). The expression of the co-stimulatory makers, CD86, CD80 and CD83, was not modified by DPTA NONOate (S3A–D Fig.). To test whether DCs were able to prime Th1, Th2 or Th17 responses, DCs treated with DPTA NONOate before LPS were washed and then co-cultured with allogenic T-cells.

**Fig 3:**
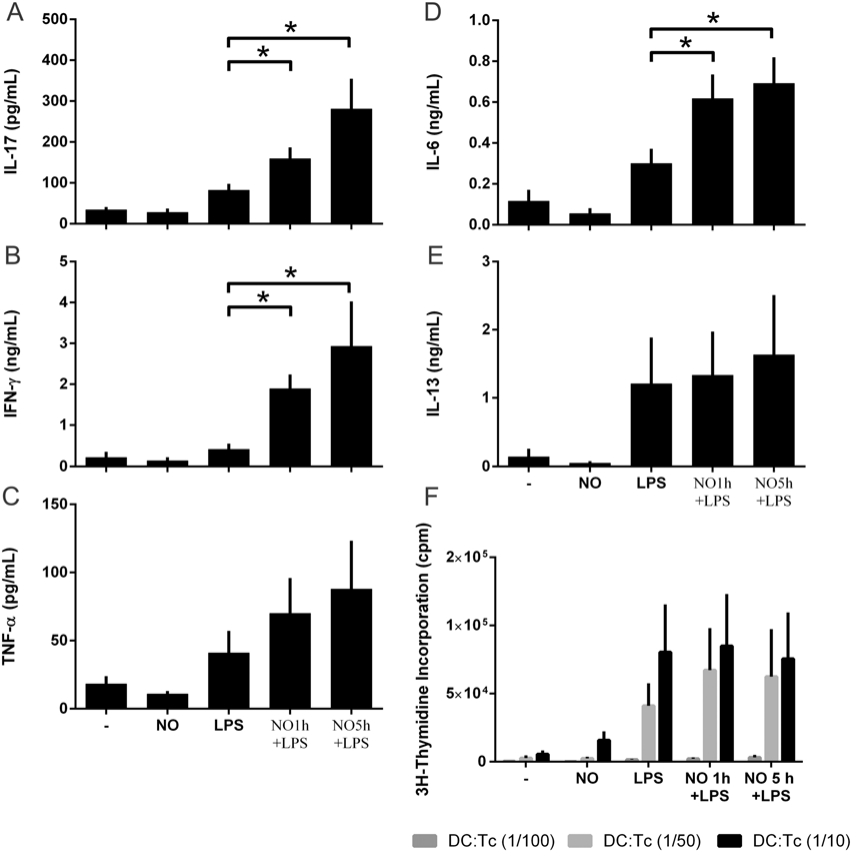
NO^.^ fails to modify the alloantigen T-cell response but enhances the release of IL-17 during a MLR. DCs were simulated in time-dependent experiments with DPTA NONOATE^.^ 0.6mM and LPS 100ng/ml. 1×10^5^ responder T-cells used for allogenic MLR assays were added to washed DCs for 6 days. A-E, Bioplex of IL-17, IFN-γ, TNF-α, IL-6, IL-13 in 6-day culture at a DC:T-cell ratio of 1:10. Data are expressed as means ±SEM of 7 independent experiments for IL-17 and 6 independent experiments for IFN-γ, TNF-α, IL-6, IL-13. F, T-cell proliferation was measured by incorporation of thymidine at DC:T-cell ratios of 1:10, 1:50 and 1:100. Data are expressed as means ±SEM from 3 independent experiments done in triplicate. *P < 0.05.

Under the co-stimulatory conditions, DPTA NONOate did not change the proportion of T-cell proliferation measured by incorporation of thymidine, whether it was added 1 or 5 hours before LPS stimulation (Fig. 3F). Interestingly, during the allo-presentation, the NO^.^-LPS-treated DCs, compared with LPS-treated DCs, were able to increase the release of IL-17 and IL-6 (Fig. 3A and D), while maintaining a strong Th1 response through the release of TNF-α with (enhanced) IFN-γ (Fig. 3B and C). Th2 responses measured by the release of IL-13 were not modulated by DCs pre-treated with NO-LPS (Fig. 3E). These results suggest that NO^.^ has an important ability to modulate DC phenotype, resulting in diverse T-helper responses during the maturation of DCs. NO^.^ did not modify the co-stimulatory molecules required for the allo-presentation to lymphocytes, but alters the kinetics of cytokine release necessary to polarize T-helper responses.

### DPTA NONOate modulates IL-1β and IL-23 release from DCs driving Th17 responses

**Fig 4:**
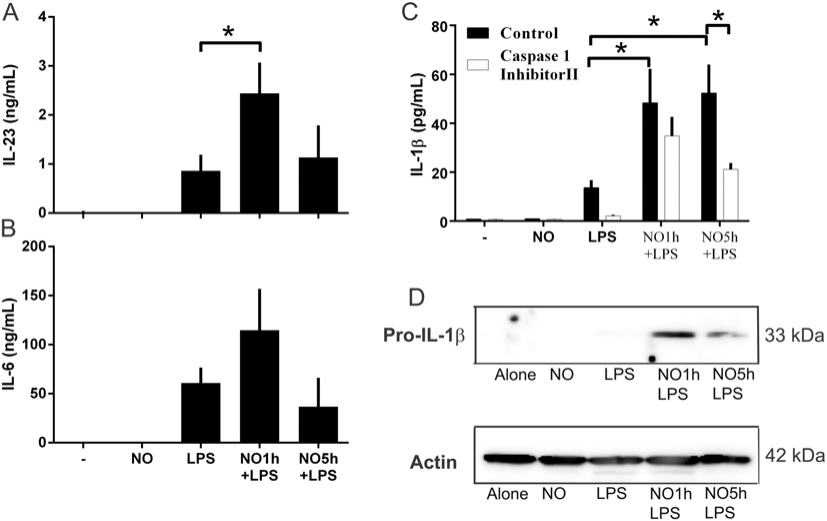
NO^.^ promotes the release of IL-23, IL-6 and IL-1β during maturation of DCs. DCs were stimulated with DPTA NONOate (0.6mM) treated DCs 1h or 5h before maturation with LPS (100ng/ml). (A) IL-23 and (B) IL-6 were analyzed in supernatants. (C) IL-1β was analyzed in supernatants, but also in DCs pre-treated with the specific caspase-I inhibitor II Ac-YVAD-CMK (50 M) for 1 h before the DPTA NONOate /LPS stimulation. (D) Cells were harvested and intracellular pro-IL-1β (blotted with a specific pro-IL1β was analyzed by Western blot. Data are expressed as means ±SEM of 6 independent experiments. *P < 0.05 or **P < 0.01.

To further characterize the role of NO^.^ in the modulation of Th17 responses, we measured the production of the additional cytokines, IL-23, IL-6, TGF-β and IL-1β by DCs treated with NO^.^ prior to addition of LPS. DPTA NONOate increased the release of IL-23 when added 1h before LPS (Fig. 4A). IL-6 was not modulated compared to LPS (Fig. 4B). The release of TGF-β was not increased compared to control conditions (unpublished data). Interestingly, IL-1βwas upregulated when compared to LPS alone, and this increase was sustained whether DPTA NONOate was used for 1 or 5 hours before LPS maturation (Fig. 4C). Different mechanisms of IL-1β release might be involved, since cell incubation in the presence of the specific caspase-1 inhibitor 1 hour before DPTA NONOate/LPS co-stimulation only inhibited the release when the cells were incubated with DPTA NONOate for 5 hours before LPS and not for 1 hour. Therefore, NO^.^ may also be involved in the expression of IL-1β, since the pro-IL-1β is enhanced intracellularly (Fig. 4D). These results suggests that NO^.^ is involved in the expression of IL-1β through at least 2 mechanisms. The expression of IL-1β is initially increased and, if NO^.^ stimulation lasts longer, the cytokine release is increased through a caspase-1 dependent mechanism (Fig. 4C). Overall, NO^.^ stimulated the release of IL-1β and IL-23, two cytokines that have been shown to be involved in the production of IL-17.

### Differential regulation of cytokines by DPTA NONOate

**Fig 5:**
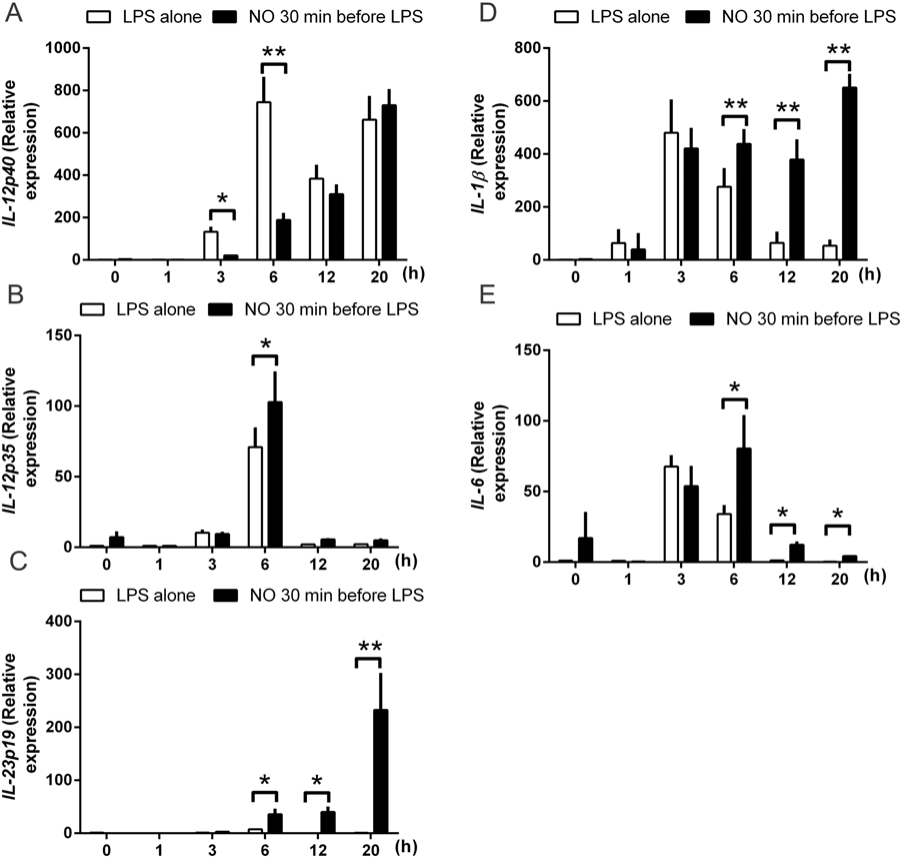
NO^.^ modulates the gene expression of IL-12, IL -23, IL-6 and IL-1β during maturation of DCs. DCs were stimulated with DPTA NONOate (0.6mM) for 30 min before maturation with LPS (100ng/ml). Cells were analyzed at the different time-points indicated. (A) *IL-12Bp40*, (B) *IL-12Ap35*, (C) *IL-23Ap19*, (D) IL-1β and(E) IL-6 levels were assessed by TaqMan real-time reverse transcription—polymerase chain reaction (RT-PCR), using β-actin as endogenous control. Data express the relative gene abundance compared to control conditions, and are shown as mean ±SEM of 4 independent experiments, done in duplicates. *P < 0.05 or **P < 0.01.

To investigate the early events that modulate cytokine expression by NO^.^ during the maturation of DCs, we measured the kinetics of the expression of mRNA encoding for *IL-12Bp40*, *IL-12Ap35*, *IL-23Ap19*, *IL-1β*and *IL-IL-6* of DCs over 20 hours (Fig. 5). DPTA NONOate added 30 min before LPS stimulation has a strong inhibitory effect on the expression of *IL-12p40* during the first 6 hours of LPS stimulation, but beyond 6 hours, the inhibition by DPTA NONOate seems to be lost (Fig. 5A). Interestingly, the expression of *IL-12p35* at 6 hours was significantly increased (Fig. 5B), but the expression over 20 hours was reduced to levels similar measured in the presence of LPS alone. The cytokines involved in Th17 polarization were highly induced. *IL-23p19* was increased after 6 hours and its expression is highly sustained for up to 20 hours when compared to LPS (Fig. 5C).

NO^.^ modulated the expression of *IL-1β*with a different time-course: the expression increased at 3 hours but was not different from the expression in LPS-stimulated DCs. The expression decreased in cells matured with LPS alone after 20 hours, unlike the cells pre-incubated with NO^.^ before LPS, where the expression was maintained (Fig. 5D). The expression of *caspase-1* was low in both LPS alone and, DPTA NONOate- and LPS-exposed DCs, and no differences over 20h were observed (unpublished data). Furthermore, IL-6 expression was also upregulated at 6 hours. Overall, these results suggest that NO^.^ plays an important modulatory role in the gene expression of cytokines, causing an initial inhibition of the expression of IL-12, followed by stimulation of the expression of cytokines involved in Th17 polarization, namely IL-23, IL-6 and IL-1β.

### IL-1β and IL-6 in IL-17 priming T-cells by DPTA NONOate

**Fig 6:**
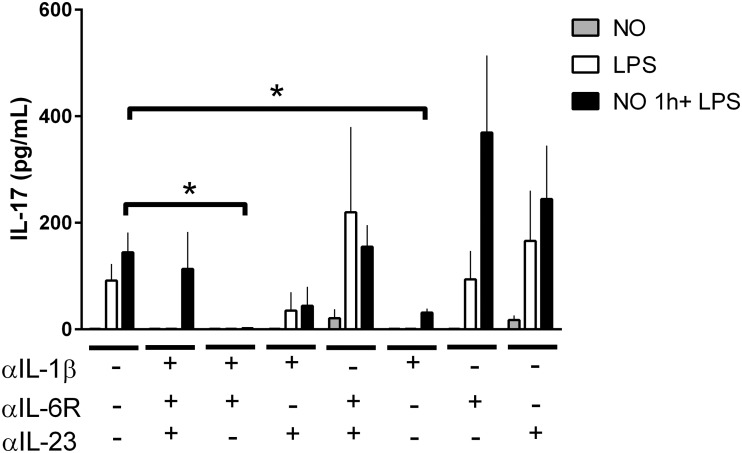
IL-1β is required to prime T-cells to produce IL-17 in a mixed lymphocyte reaction condition. Production of IL-17 by T-cells primed for 6 days with allogenic DCs at a DC:T-cell ratio of 1:10. As described in Materials and Methods, DCs were simulated with DPTA NONOate (0.6mM) 1h before LPS (100ng/ml) stimulation. During the MLR, cells were cultured in the presence of neutralizing antibodies to IL-23, IL-1β and IL-6R. Data are expressed as mean ±SEM of 3 independent experiments. *P < 0.05.

To further document the function of NO^.^ and hence the role of IL-23, IL-1β and IL-6 in the ability of LPS-DCs to induce Th17 responses, we evaluated the effects of anti-IL-23, anti-IL-1β and anti-IL-6 receptor (IL-6R) neutralizing antibodies. Treatment with neutralizing antibodies to IL-1β or both IL-1β and IL-6R abolished the release of IL-17 by T-cells induced by LPS-activated DCs, whereas neutralization of IL-23 did not affect the release (Fig. 6). These results indicate that the ability of NO^.^ to induce IL-17 release is critically dependent on the production of IL-1β and on the synergistic effect of IL-6 with IL-1, but is not dependent on IL-23 or IL-6 alone.

### Carboxy-PTIO specifically inhibits the DPTA NONOate induction of IL-1β and IL-23

**Fig 7:**
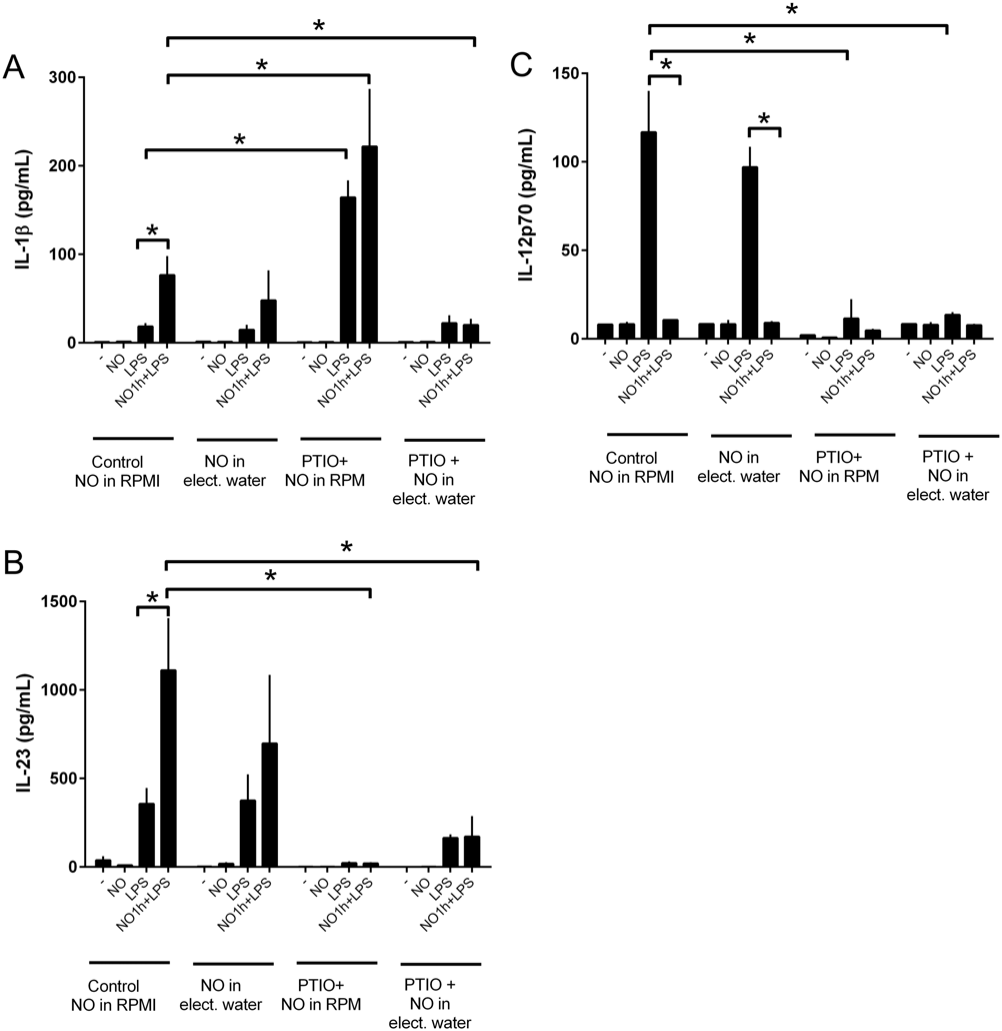
Carboxy-PTIO inhibits the DPTA NONOate induction of IL-1β and IL-23. DCs were stimulated with Carboxy-PTIO (5μM) 30 min before treatment with DPTA NONOATE (0.6mM in RPMI or electrolyzed water (EW)). After 1h stimulation cells were maturated with LPS (100ng/ml). The secretion of cytokines was analyzed using the Luminex system: (A) IL-1β, (B) IL-23 and (C) IL-12p70. Data are expressed as means ±SEM of 3 independent experiments. *P < 0.05.

In order to determine the specificity of NO, for the experiments, Carboxy-PTIO, was added to immature DC 30 min prior DPTA NONOate treatment. After 1 h incubation cells were matured with LPS. As shown in Fig. 7, the release of IL-23 induced by LPS or NO-LPS stimulation was markedly blocked by the addition of carboxy-PTIO (Fig. 7B). However, carboxy-PTIO markedly boosted the induction of IL-1β in both LPS and DPTA-Nonoate-LPS treatment (Fig. 7A). Carboxy-PTIO has been recognized as a specific NO scanvenger which directly oxidizes NO to form NO^-^_2_ and promote N_2_O_3_ formation [23]. Thus, the generation of these NO oxidative products may be involved in the IL-1β pathway activation, making difficult to determine the specificity of NO. In order to reduce active oxygen species generated during the co-stimulation with Carboxy-PTIO and DPTA NONOate, DPTA NONOate was dissolved in electrolyzed water (EW), which potently scavenges active oxygen species [22]. DPTA NONOate in EW release NO in a similar manner as in RPMI (S4 Fig). DPTA NONOate was added to DC pre treated or not with carboxy-PTIO and then matured with LPS. As a control 6 μL of EW was added to DC or LPS-DCs condition. DPTA NONOate in EW slightly decreases the release of IL-1β and IL-23 as compared to control conditions and maintains the inhibition of IL-12. Interestingly, in the presence of EW, carboxy-PTIO specifically inhibited the release of IL-1β and IL-23 without modifying the LPS response (Fig. 7A and B). In contrast, carboxy-PTIO in EW condition does not overcome the inhibitory effect of NO on the release of IL-12. Furthermore, carboxy-PTIO suppressed the LPS-release of IL-12 (Fig. 7C) suggesting that another unspecific effect of PTIO, which cannot be counteracted by EW, may act on the IL-12 pathway.

## Discussion

We have analysed the role of NO^.^ *in* modulating DC response to LPS. The most important novel finding of our work is that NO^.^ changes the pattern of cytokine expression and release by LPS-matured DCs, predominantly involved in the priming of IL-17-producing T-cells such as Th-17 cells, through the release of IL-1β, thereby highlighting the role of NO^.^ in sustaining inflammation during infection. The timing of NO^.^ administration, prior to the maturation of DCs, as well as the concentrations of NO^.^ in activating DCs were important factors that determined the different mechanisms that NO^.^ can have recourse to in the modulation of the T-cell response. We modelled our experiments on the *in vivo* situation, where the generation of NO^.^ prior to any injurious stimulus to the airway epithelium is akin to the situation in the asthmatic or COPD patient in which fully-differentiated immature DCs are exposed to NO^.^ on arrival in the subepithelial tissue. The question we asked was how these DCs might respond to an infection and thereby induce a different T-helper polarization. LPS has been used to model bacterial inflammation, particularly gram-negative bacteria since LPS is the most abundant component within the cell wall of Gram-negative bacteria which can be a major cause of exacerbations in lung diseases such as COPD.

We found that the response was dependent on the concentration of NO. Thus, high concentrations of NO-donor (1mM) prior to LPS maturation directed the immune response towards an anti-inflammatory pattern by reducing IL-12 release, while maintaining IL-10 and TNF-α production.

The timing of the NO exposure was also influential. The NO-donor (0.6mM) added prior to maturation resulted in a significant inhibition of IL-12, which can be partially explained by the specific down-regulation of the *IL-12p40* gene transcription observed earlier on at 1–6 hours, but post-transcriptional factors may also be involved, since *IL-12p40* gene transcription continues after 12 hours. The intracellular mechanism regulating the secretion of bioactive IL-12 may involve a transient modulation of *IL-12p35* gene expression as reported previously [24], whereas after 6 hours of co-stimulation, NO^.^ increased the transcription of *IL-23p19*, which, together with *IL-12p40* gene transcript, led to the generation of IL-23. IL-23 is indeed increased in the supernatant after 1h of incubation, suggesting the occurrence of post-transcriptional events favouring the assembly of IL-12p40 with IL-23p19, particularly at earlier time-points.

Our detailed time-course and concentration-effect studies may also help explain some of the conflicting reports of the effects of NO^.^ on DCs in the literature. On the one hand, NO^.^ increased the ability of DCs to activate T-cells through the increased release of IL-12, which is in contrast to those groups who have demonstrated that NO^.^ is able to inhibit IL-12 transcription and NF-κB activation during LPS maturation of DCs [7–9].

There has been also conflicting reports on the role of NO in the induction of IL-1β and their release [25]. We can speculate that differences can be observed not only between macrophages and dendritic cells, but also between murine and human cells. In fact in our laboratory in the context of analysing the role of *Salmonella* virulence factor SipB, the activation and release of IL-18 in human dendritic cells was shown to be mediated in a caspase-1 independent mechanism [26]. This observation was in contrast to murine macrophages in which the induction of the release of IL-18 was shown to be mediated by a caspase-1 dependent mechanism [27, 28].

The specificity of NO in the release of IL-1β and IL-23 was demonstrated using carboxy-PTIO. The release of IL-23 has shown to be sensitive to NO. However, carboxy-PTIO boosted the induction of IL-1β. It is known that PTIO scavenge NO to form NO^-^_2_. NO^-^_2_ can react with the remaining NO to promote N_2_O_3_ formation [23, 29]. This oxidative stress generated with exogenous NO (DPTA-Nonoate) or endogenous NO induced by LPS [30] with carboxy-PTIO might be the factor that boosts the release of IL-1βThis effect has been reported in other cell models such as monocytes [29] might be the factor that boosts the release of IL-1β. This result is very interesting because it demonstrates the sensibility of IL-1β pathway to NO and NO-derivate species. It is not yet clear the mechanism by which carboxy-PTIO maintains a strong suppression of IL-12, we can speculate that the IL-12 activation pathway is sensitive to NO and NO-derivate species; however, in spite of the large spectrum of electrolyzed water (EW) to scavenge oxidative species that can react with NO, EW was not sufficient to antagonized the inhibitory effect of PTIO.

We also showed that NO^.^ is able to increase IL-6 gene transcription, while maintaining the transcription of IL-1β. In contrast, TGF-β was not modulated by NO^.^. The production of IL-1β and IL-6 but not IL-12 induced by NO^.^ can enhance Th17 differentiation [31, 32]. In our mixed lymphocyte reaction, IL-6 was produced, but interestingly only the inhibition of IL-6 in combination with IL-1β or of IL-1βalone, was critically important for the polarization of Th17, suggesting that in humans IL-1β may play a most relevant role in the production of IL-17, and IL-6 may synergise with the stimulatory activity of IL-1β. We strongly believe that the T-cells induced in our mixed lymphocyte reaction are indeed of a Th17 lineage and not of γδT cells for 2 reasons. Firstly, γδT cells are involved largely in innate-like response, expressing TLR-1, TLR-2, and dectin-1, and they do not require MHC presentation to recognize peptides, though the pathogen itself or its metabolites, epithelial stress signals and cytokines such as IL-23 and IL1β are required to cause optimal release of IL-17 [33–35]. Thus, the priming of γδT with allogenic matured DCs may be insufficient to induce the secretion of IL-17. Furthermore, γδT cells do not express IL-6R and IL-6 was shown to be dispensable for the activation of IL-17 in these cells [36].

NO^.^ could favour the priming of both Th17 and Th1 responses without modifying Th2 responses. We can hypothesize that NO^.^ could be more involved in increasing the neutrophilic aspects of inflammation but may not modulate Th2 polarization. It is difficult to explain why NO^.^ inhibited the release of IL-12 without inhibiting Th1 responses in the mixed lymphocyte reaction. However, there is increased evidence that the polarization of Th1, Th17 and Tregs is a result of the interaction of different signals such as IFN-γ, IL-6, or TGF-β and the balance of these interactions will favour the expression of T-bet or ROR-γt, and hence, the final outcome of the T-helper differentiation [10, 31, 37]. Thus, IFN-γ can increase the expression of T-bet for Th1 polarization, but the presence of TGF-β is able to abolish the expression T-bet [10, 31]. IL-6 and TGF-βwill favour a Th17 polarization by increasing the expression of ROR-γt, but TGF-β alone will favour the development of Tregs [10]. Therefore, Th17 and Th1 cells can be found to some extent under the same culture conditions [10, 31]. Lee and co-workers have demonstrated that NO^.^ is a co-factor that can directly regulate differentiation signals on T-helper cells, particularly reinforcing IFN-γR signalling of T cells, thus favouring Th1 development. Furthermore, in the presence of TGF-β the relative levels of NO^.^ with regard to the levels of IL-6 was the key combination that determined Th1 versus Th17 differentiation [10].

One important aspect at this point are the different outcomes observed between human and mouse studies. In support of our results, it has also been demonstrated by other groups that NO^.^ produced by human myeloid-derived suppressor cells selectively increases the expression of RORγt and hence IL-17A production in naïve and memory T cells [38]. These results were confirmed using exogenous NO^.^ donor [38]. In contrast, in mouse models, endogenous as well as exogenous NO^.^ has been shown to be a suppressor of Th17 polarization not only through a direct effect of NO on the release of IL-17 observed in an iNOS deficient model, but also by the induction of a regulatory T cell expressing CD4^+^CD25^+^FoxP3^-^GITR^+^CD27^+^ able to suppress the induction of Th17 cells [11, 39]. It is important to note that inhibitory properties of NO^.^ have been observed in the framework of T cell activation supplemented with Th17-driving cytokines and not in its capacity to modulate the Th17 cytokine pattern on DCs. As was discussed previously, the cytokine environment importantly determines the final T-helper outcome. TGF-β seems to be the key difference between humans and mice regarding Th17 polarization. In mouse models, TGFβ can be placed as a central modulator in which NO^.^ is able to reprogram its action and, in synergy with IL-6, a direct expansion of the Th17 lineage [10]. Thus, the protective role observed *in vivo*, as shown in a model of experimental autoimmune encephalomyelitis (EAE) [40], could reflect also an inhibitory role of NO^.^ on TGF-β and not a direct role of NO^.^ on IL-17 transcription. This is in contrast with what is observed in humans, where TGF-β per se inhibits RORτ expression and Th17 polarization [31], suggesting that other NO^.^-regulatory mechanisms may be involved. These discrepancies highlight the great variability of NO^.^ action not only in turning-on/off cascades but also in modulating its action depending on the prevailing cytokine environment. For example, IL-1β and the synergistic effect of IL-6 with IL1β, may tip the balance in favour of IL-17 production in the presence of human DCs without TGF-β modulation.

What we consider to be valuable in our model is the fact that in our mixed lymphocyte response experiments, NO^.^ has not been added to the co-cultures. Thus, the effect of NO^.^ demonstrated here represents the initial modulation of DCs and at a distance the capacity to modulate a T-helper response, without modulating the alloantigen-presenting capacity of DCs (unlike superoxide anions as we have shown previously [41]). In contrast to previous studies where monocytes and conventional DCs, but not monocyte-derived DCs, efficiently primed IL-17-producing T cells [31], we have used monocyte-derived DCs and we show that NO^.^ was the key co-factor to reprogram these cells to induce the profile required for Th17 polarization. We hypothesize that *in vivo* NO^.^ produced by the epithelium will be able to reprogram DCs when these cells are recalled to the submucosal tissue, since the epithelium produced the largest quantities of NO^.^ [3]. Other sources of NO^.^ such as that derived from TipDCs (TNF-α/inducible nitric oxide synthase (iNOS)-producing DCs) not only in the airways and lung parenchyma but also in lymphoid tissues deserve further exploration [42].

The role played by IL-17 in lung diseases such as asthma and COPD via the regulation of neutrophil and monocyte recruitment is becoming established [43, 44]. IL-17 has been also associated with ischemia-reperfusion injury, and is increased in patients with bronchiolitis obliterans syndrome, indicating a potential role in lung rejection [45]. Therefore, NO^.^ may play a critical role in lung diseases by modulating the expression of IL-17 through the modulation of IL-1β. To what extent IL-17 is beneficial or harmful is not completely unravelled. However, therapies involving complete abrogation of NO^.^ will not be useful when a Th17 response is required e.g. against a fungal or chronic infection such as *M*. *tuberculosis*. In recent years, it has been suggested that the use of antioxidants, and particularly NO^.^ inhibitors, could be useful in the treatment of lung inflammatory conditions such as asthma. However, results have been disappointing so far, asin some cases, NO^.^ inhibitors have shown to worsen disease control, which suggests a protective role of ROS [46]. On the other hand, therapies involving partial reduction of NO^.^ in combination with/or blocking IL-1β may result in effective abrogation of neutrophil recruitment. Further studies using chronic inflammatory models reflecting neutrophilic asthma or COPD will be needed to better assess the role of NO^.^

In conclusion, we have demonstrated that in human DCs, NO^.^ potently modulates DC response to LPS, predominantly by increasing the expression and release of IL-1β and consequently the priming of IL-17-producing T-cells such as Th-17 cells. Therapies leading to partial reduction of NO^.^ or such as by blocking IL-1β should be considered [47].

## Supporting Information

S1 Fig LPS protects cells from apoptosis and necrosis. DCs were stimulated with DPTA NONOate (0.6mM) 10 min and 5h before LPS maturation with LPS (100ng/ml) (NO10’+LPS); NO5h+LPS). For control conditions, DCs were treated with 1mM or 0.6mM DPTA NONOate. Apoptosis and necrosis were determined by flow cytometry with annexin-V-FITC and PI, respectively. N+A represents the percentage of necrosis plus apoptosis. The results shown are representative of two independent experiments.
(TIF)

S2 Fig Release of IL-12p40 by NO^.^ during maturation of DCs. DCs were stimulated with DPTA NONOate (0.6mM) 1h or 5h before maturation with LPS (100ng/ml) (NO10’+LPS); NO5h+LPS). The secretion of IL-12p40 was analyzed using a luminex system. Data are expressed as mean ± SEM of 3 independent experiments. *P < 0.05.
(TIF)

S3 Fig NO^.^ failed to change expression of maturation markers such as CD86, 83 and 80. DCs were stimulated with DPTA NONOate (0.6mM) 10 min and 5h before maturation with LPS. (A and B) CD86, (C) CD83, and (D) CD80. (B) Additionally, DCs were stimulated with different concentrations of DPTA NONOate 10 minutes before maturation with LPS. Data are expressed as mean ± SEM of 5 independent experiments for the time-dependent analysis and 3 independent experiments for the concentration dependent analysis. *P < 0.05.
(TIF)

S4 Fig Fluorescence emission of DAF-FM in solutions containing 0.6mM of DPTA-Nonoate. For the experiments DPTA NONOATE (0.6mM) was resuspended in RPMI or electrolized water (EW). Fluorescence emission intensity of DAF-FM (5μM) was measured by a 96-well plate reader. The results shown are representative of two independent experiments.
(TIF)

## References

[1] Banchereau J, Briere F, Caux C, Davoust J, Lebecque S, Liu YJ, et al Immunobiology of dendritic cells. Annual review of immunology. 2000;18:767–811. 10837075 10.1146/annurev.immunol.18.1.767

[2] Roelen DL, van den Boogaardt DE, van Miert PP, Koekkoek K, Offringa R, Claas FH. Differentially modulated dendritic cells induce regulatory T cells with different characteristics. Transpl Immunol. 2008;19(3–4):220–8. 10.1016/j.trim.2008.05.002 18639635

[3] Xu W, Zheng S, Dweik RA, Erzurum SC. Role of epithelial nitric oxide in airway viral infection. Free radical biology & medicine. 2006;41(1):19–28. 16781449 10.1016/j.freeradbiomed.2006.01.037 PMC7127628

[4] Lim KG, Mottram C. The use of fraction of exhaled nitric oxide in pulmonary practice. Chest. 2008;133(5):1232–42. 10.1378/chest.07-1712 18460522

[5] Barnes PJ. Reduced histone deacetylase in COPD: clinical implications. Chest. 2006;129(1):151–5. 16424426 10.1378/chest.129.1.151

[6] Brindicci C, Ito K, Resta O, Pride NB, Barnes PJ, Kharitonov SA. Exhaled nitric oxide from lung periphery is increased in COPD. Eur Respir J. 2005;26(1):52–9. 15994389 10.1183/09031936.04.00125304

[7] Paolucci C, Burastero SE, Rovere-Querini P, De Palma C, Falcone S, Perrotta C, et al Synergism of nitric oxide and maturation signals on human dendritic cells occurs through a cyclic GMP-dependent pathway. Journal of leukocyte biology. 2003;73(2):253–62. 12554802 10.1189/jlb.0902447

[8] Xiong H, Zhu C, Li F, Hegazi R, He K, Babyatsky M, et al Inhibition of interleukin-12 p40 transcription and NF-kappaB activation by nitric oxide in murine macrophages and dendritic cells. The Journal of biological chemistry. 2004;279(11):10776–83. 14679201 10.1074/jbc.M313416200

[9] Corinti S, Pastore S, Mascia F, Girolomoni G. Regulatory role of nitric oxide on monocyte-derived dendritic cell functions. J Interferon Cytokine Res. 2003;23(8):423–31. 13678430 10.1089/107999003322277838

[10] Lee SW, Choi H, Eun SY, Fukuyama S, Croft M. Nitric oxide modulates TGF-beta-directive signals to suppress Foxp3+ regulatory T cell differentiation and potentiate Th1 development. J Immunol. 2011;186(12):6972–80. 10.4049/jimmunol.1100485 21555530 PMC3113707

[11] Niedbala W, Besnard AG, Jiang HR, Alves-Filho JC, Fukada SY, Nascimento D, et al Nitric oxide-induced regulatory T cells inhibit Th17 but not Th1 cell differentiation and function. J Immunol. 2013;191(1):164–70. 10.4049/jimmunol.1202580 23720815 PMC3785138

[12] Aujla SJ, Chan YR, Zheng M, Fei M, Askew DJ, Pociask DA, et al IL-22 mediates mucosal host defense against Gram-negative bacterial pneumonia. Nature medicine. 2008;14(3):275–81. 10.1038/nm1710 18264110 PMC2901867

[13] Nanzer AM, Chambers ES, Ryanna K, Richards DF, Black C, Timms PM, et al Enhanced production of IL-17A in patients with severe asthma is inhibited by 1alpha,25-dihydroxyvitamin D3 in a glucocorticoid-independent fashion. The Journal of allergy and clinical immunology. 2013;132(2):297–304 e3 10.1016/j.jaci.2013.03.037 23683514

[14] Murdoch JR, Lloyd CM. Resolution of allergic airway inflammation and airway hyperreactivity is mediated by IL-17-producing {gamma}{delta}T cells. American journal of respiratory and critical care medicine. 2010;182(4):464–76. 10.1164/rccm.200911-1775OC 20413629 PMC2937240

[15] Chang Y, Nadigel J, Boulais N, Bourbeau J, Maltais F, Eidelman DH, et al CD8 positive T cells express IL-17 in patients with chronic obstructive pulmonary disease. Respiratory research. 2011;12:43 10.1186/1465-9921-12-43 21477350 PMC3082241

[16] Kaiser L, Aubert JD, Pache JC, Deffernez C, Rochat T, Garbino J, et al Chronic rhinoviral infection in lung transplant recipients. American journal of respiratory and critical care medicine. 2006;174(12):1392–9. 17008640 10.1164/rccm.200604-489OC

[17] Chun E, Lee SH, Lee SY, Shim EJ, Cho SH, Min KU, et al Toll-like receptor expression on peripheral blood mononuclear cells in asthmatics; implications for asthma management. J Clin Immunol. 2010;30(3):459–64. 10.1007/s10875-009-9363-z 20072849

[18] Searing DA, Rabinovitch N. Environmental pollution and lung effects in children. Curr Opin Pediatr. 2011;23(3):314–8. 10.1097/MOP.0b013e3283461926 21467938

[19] Obregon C, Rothen-Rutishauser B, Gerber P, Gehr P, Nicod LP. Active uptake of dendritic cell-derived exovesicles by epithelial cells induces the release of inflammatory mediators through a TNF-alpha-mediated pathway. The American journal of pathology. 2009;175(2):696–705. 10.2353/ajpath.2009.080716 19628765 PMC2715287

[20] Mentzer SJ, Guyre PM, Burakoff SJ, Faller DV. Spontaneous aggregation as a mechanism for human monocyte purification. Cellular immunology. 1986;101(2):312–9. 3757046 10.1016/0008-8749(86)90144-9

[21] Sallusto F, Lanzavecchia A. Efficient presentation of soluble antigen by cultured human dendritic cells is maintained by granulocyte/macrophage colony-stimulating factor plus interleukin 4 and downregulated by tumor necrosis factor alpha. J Exp Med. 1994;179(4):1109–18. 8145033 10.1084/jem.179.4.1109 PMC2191432

[22] Shirahata S, Kabayama S, Nakano M, Miura T, Kusumoto K, Gotoh M, et al Electrolyzed-reduced water scavenges active oxygen species and protects DNA from oxidative damage. Biochemical and biophysical research communications. 1997;234(1):269–74. 9169001 10.1006/bbrc.1997.6622

[23] Amano F, Noda T. Improved detection of nitric oxide radical (NO.) production in an activated macrophage culture with a radical scavenger, carboxy PTIO and Griess reagent. FEBS letters. 1995;368(3):425–8. 7543422 10.1016/0014-5793(95)00700-j

[24] Goriely S, Molle C, Nguyen M, Albarani V, Haddou NO, Lin R, et al Interferon regulatory factor 3 is involved in Toll-like receptor 4 (TLR4)- and TLR3-induced IL-12p35 gene activation. Blood. 2006;107(3):1078–84. 16219795 10.1182/blood-2005-06-2416

[25] Kim YM, Talanian RV, Li J, Billiar TR. Nitric oxide prevents IL-1beta and IFN-gamma-inducing factor (IL-18) release from macrophages by inhibiting caspase-1 (IL-1beta-converting enzyme). J Immunol. 1998;161(8):4122–8. 9780184

[26] Dreher D, Kok M, Obregon C, Kiama SG, Gehr P, Nicod LP. Salmonella virulence factor SipB induces activation and release of IL-18 in human dendritic cells. Journal of leukocyte biology. 2002;72(4):743–51. 12377944

[27] Obregon C, Dreher D, Kok M, Cochand L, Kiama GS, Nicod LP. Human alveolar macrophages infected by virulent bacteria expressing SipB are a major source of active interleukin-18. Infection and immunity. 2003;71(8):4382–8. 12874316 10.1128/IAI.71.8.4382-4388.2003 PMC166028

[28] Hersh D, Monack DM, Smith MR, Ghori N, Falkow S, Zychlinsky A. The Salmonella invasin SipB induces macrophage apoptosis by binding to caspase-1. Proceedings of the National Academy of Sciences of the United States of America. 1999;96(5):2396–401. 10051653 10.1073/pnas.96.5.2396 PMC26795

[29] Turpaev K, Bouton C, Diet A, Glatigny A, Drapier JC. Analysis of differentially expressed genes in nitric oxide-exposed human monocytic cells. Free radical biology & medicine. 2005;38(10):1392–400. 15855057 10.1016/j.freeradbiomed.2005.02.002

[30] Matsuzawa A, Saegusa K, Noguchi T, Sadamitsu C, Nishitoh H, Nagai S, et al ROS-dependent activation of the TRAF6-ASK1-p38 pathway is selectively required for TLR4-mediated innate immunity. Nature immunology. 2005;6(6):587–92. 15864310 10.1038/ni1200

[31] Acosta-Rodriguez EV, Napolitani G, Lanzavecchia A, Sallusto F. Interleukins 1beta and 6 but not transforming growth factor-beta are essential for the differentiation of interleukin 17-producing human T helper cells. Nature immunology. 2007;8(9):942–9. 17676045 10.1038/ni1496

[32] Wilson NJ, Boniface K, Chan JR, McKenzie BS, Blumenschein WM, Mattson JD, et al Development, cytokine profile and function of human interleukin 17-producing helper T cells. Nature immunology. 2007;8(9):950–7. 17676044 10.1038/ni1497

[33] Fenoglio D, Poggi A, Catellani S, Battaglia F, Ferrera A, Setti M, et al Vdelta1 T lymphocytes producing IFN-gamma and IL-17 are expanded in HIV-1-infected patients and respond to Candida albicans. Blood. 2009;113(26):6611–8. 10.1182/blood-2009-01-198028 19395673

[34] Vantourout P, Hayday A. Six-of-the-best: unique contributions of gammadelta T cells to immunology. Nature reviews. 2013;13(2):88–100. 10.1038/nri3384 23348415 PMC3951794

[35] Mills KH, Dungan LS, Jones SA, Harris J. The role of inflammasome-derived IL-1 in driving IL-17 responses. Journal of leukocyte biology. 2013;93(4):489–97. 10.1189/jlb.1012543 23271701

[36] Lochner M, Peduto L, Cherrier M, Sawa S, Langa F, Varona R, et al In vivo equilibrium of proinflammatory IL-17+ and regulatory IL-10+ Foxp3+ RORgamma t+ T cells. J Exp Med. 2008;205(6):1381–93. 10.1084/jem.20080034 18504307 PMC2413035

[37] Acosta-Rodriguez EV, Rivino L, Geginat J, Jarrossay D, Gattorno M, Lanzavecchia A, et al Surface phenotype and antigenic specificity of human interleukin 17-producing T helper memory cells. Nature immunology. 2007;8(6):639–46. 17486092 10.1038/ni1467

[38] Obermajer N, Wong JL, Edwards RP, Chen K, Scott M, Khader S, et al Induction and stability of human Th17 cells require endogenous NOS2 and cGMP-dependent NO signaling. J Exp Med. 2013;210(7):1433–445. 23797095 10.1084/jem.20121277 PMC3698515

[39] Jianjun Y, Zhang R, Lu G, Shen Y, Peng L, Zhu C, et al T cell-derived inducible nitric oxide synthase switches off Th17 cell differentiation. J Exp Med. 2013;210(7):1447–62. 23797094 10.1084/jem.20122494 PMC3698516

[40] Nath N, Morinaga O, Singh I. S-nitrosoglutathione a physiologic nitric oxide carrier attenuates experimental autoimmune encephalomyelitis. J Neuroimmune Pharmacol. 2010;5(2):240–51. 10.1007/s11481-009-9187-x 20091246 PMC2965418

[41] Kantengwa S, Jornot L, Devenoges C, Nicod LP. Superoxide anions induce the maturation of human dendritic cells. American journal of respiratory and critical care medicine. 2003;167(3):431–7. 12554628 10.1164/rccm.200205-425OC

[42] Serbina NV, Jia T, Hohl TM, Pamer EG. Monocyte-mediated defense against microbial pathogens. Annual review of immunology. 2008;26:421–52. 10.1146/annurev.immunol.26.021607.090326 18303997 PMC2921669

[43] Vanaudenaerde BM, Verleden SE, Vos R, De Vleeschauwer SI, Willems-Widyastuti A, Geenens R, et al Innate and adaptive interleukin-17-producing lymphocytes in chronic inflammatory lung disorders. American journal of respiratory and critical care medicine. 2011;183(8):977–86. 10.1164/rccm.201007-1196PP 21097694

[44] Alcorn JF, Crowe CR, Kolls JK. TH17 cells in asthma and COPD. Annual review of physiology. 2010;72:495–516. 10.1146/annurev-physiol-021909-135926 20148686

[45] Saini D, Weber J, Ramachandran S, Phelan D, Tiriveedhi V, Liu M, et al Alloimmunity-induced autoimmunity as a potential mechanism in the pathogenesis of chronic rejection of human lung allografts. J Heart Lung Transplant. 2011;30(6):624–31. 10.1016/j.healun.2011.01.708 21414808 PMC3091959

[46] Mulrennan SA, Redington AE. Nitric oxide synthase inhibition: therapeutic potential in asthma. Treatments in respiratory medicine. 2004;3(2):79–88. 15182209 10.2165/00151829-200403020-00002

[47] Grant RW, Dixit VD. Mechanisms of disease: inflammasome activation and the development of type 2 diabetes. Front Immunol. 2013;4(50):1–10. 23483669 10.3389/fimmu.2013.00050 PMC3592198

