# Supplementary figures and images for: Nitric Oxide Sustains IL-1β Expression in Human Dendritic Cells Enhancing Their Capacity to Induce IL-17–Producing T-Cells
Source: PLoS One. 2015 Apr 8;10(4):e0120134. doi: 10.1371/journal.pone.0120134 (PMC4390375; doi:10.1371/journal.pone.0120134)

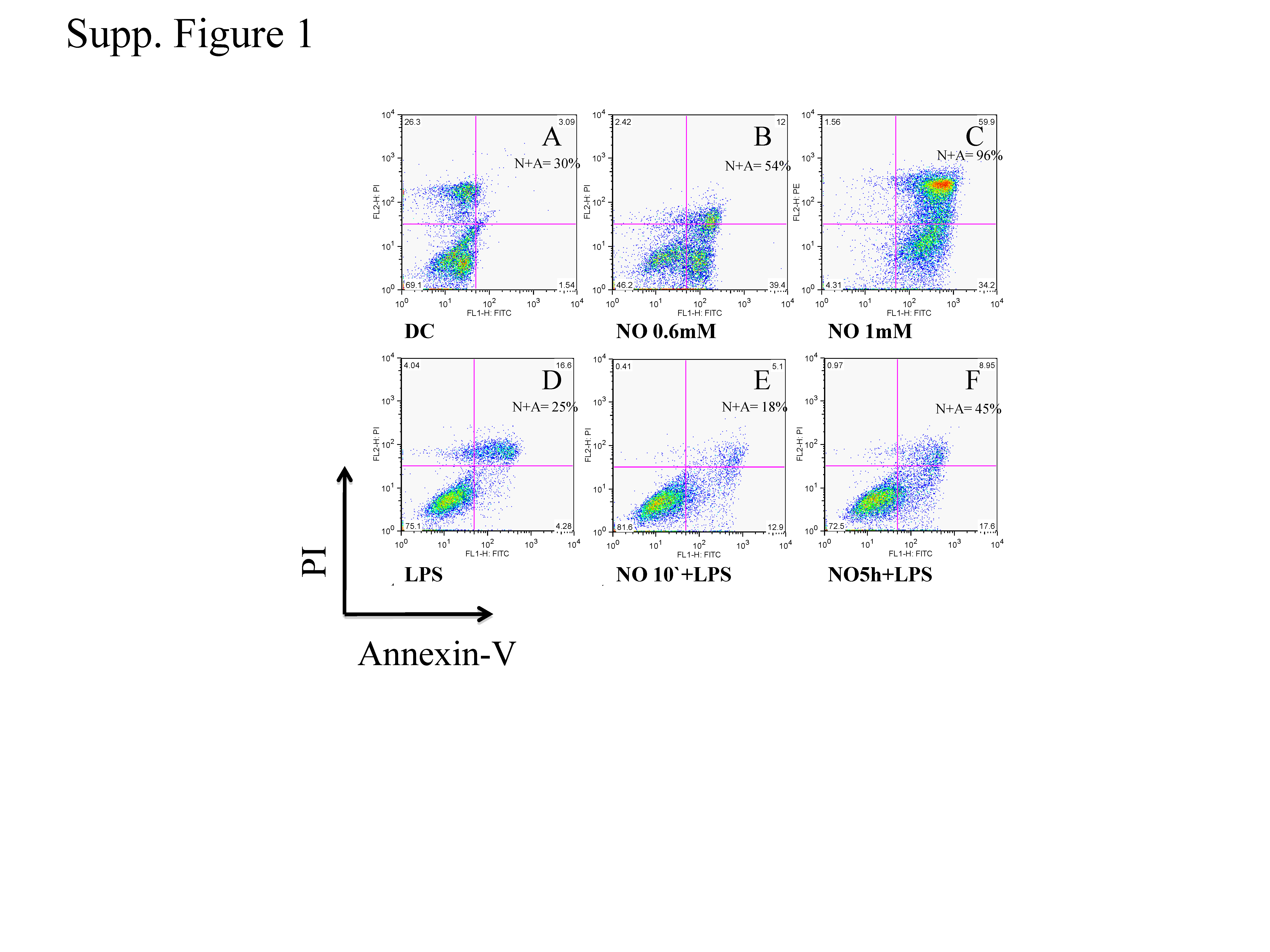

Supplement: S1 Fig — DCs were stimulated with DPTA NONOate (0.6mM) 10 min and 5h before LPS maturation with LPS (100ng/ml) (NO10’+LPS); NO5h+LPS). For control conditions, DCs were treated with 1mM or 0.6mM DPTA NONOate. Apoptosis and necrosis were determined by flow cytometry with annexin-V-FITC and PI, respectively. N+A represents the percentage of necrosis plus apoptosis. The results shown are representative of two independent experiments. (TIF) [file pone.0120134.s001.tif]

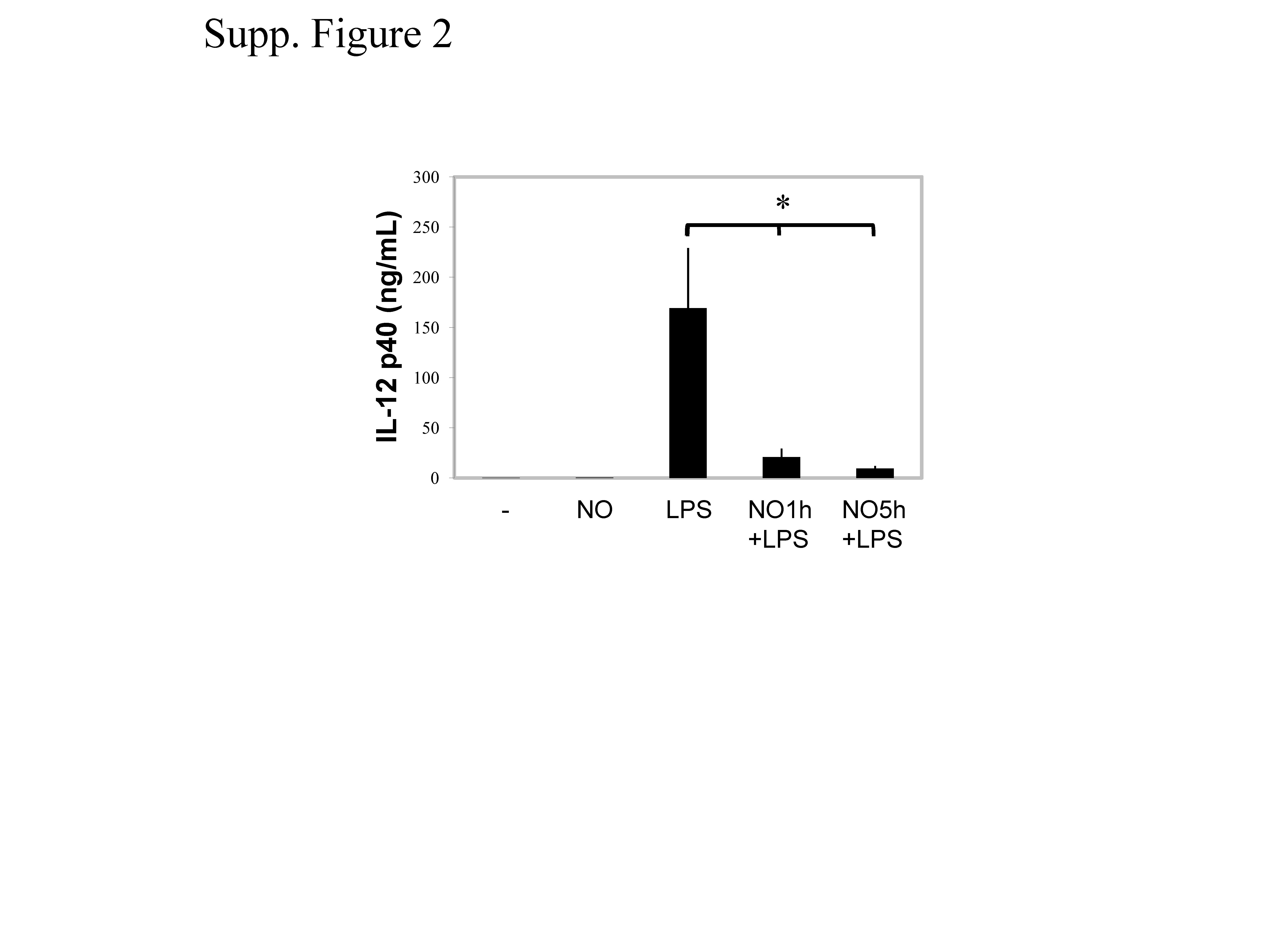

Supplement: S2 Fig — DCs were stimulated with DPTA NONOate (0.6mM) 1h or 5h before maturation with LPS (100ng/ml) (NO10’+LPS); NO5h+LPS). The secretion of IL-12p40 was analyzed using a luminex system. Data are expressed as mean ± SEM of 3 independent experiments. *P < 0.05. (TIF) [file pone.0120134.s002.tif]

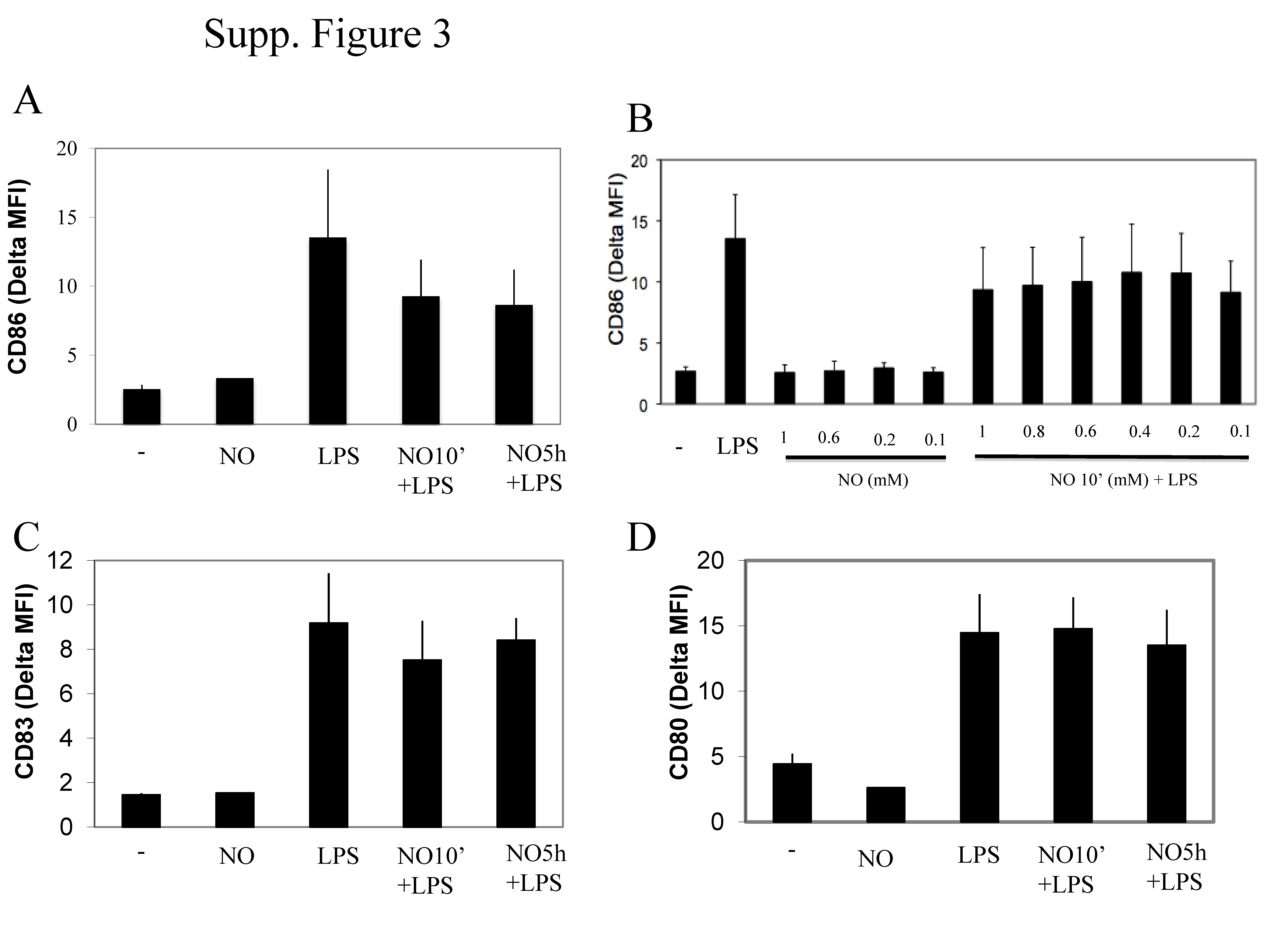

Supplement: S3 Fig — DCs were stimulated with DPTA NONOate (0.6mM) 10 min and 5h before maturation with LPS. (A and B) CD86, (C) CD83, and (D) CD80. (B) Additionally, DCs were stimulated with different concentrations of DPTA NONOate 10 minutes before maturation with LPS. Data are expressed as mean ± SEM of 5 independent experiments for the time-dependent analysis and 3 independent experiments for the concentration dependent analysis. *P < 0.05. (TIF) [file pone.0120134.s003.tif]
